# The incorporation of HIV self-testing as an exclusive option among men who have sex with men in Spain: results of an online cross-sectional study

**DOI:** 10.1186/s12889-020-09976-9

**Published:** 2020-12-04

**Authors:** J. Hoyos, J. M. Guerras, K. Koutentakis, L. de la Fuente, J. Pulido, L. Sordo, F. Vallejo, M. J. Belza

**Affiliations:** 1 Department of Public Health and Maternal and Child Health, Complutense University of Madrid, Madrid, Spain; 2 Consortium for Biomedical Research in Epidemiology & Public Health (CIBERESP), Madrid, Spain; 3 National Center of Epidemiology, Carlos III Health Institute, Madrid, Spain; 4 Department of Biostatistics and Epidemiology, National School of Health, Carlos III Health Institute, Madrid, Spain

**Keywords:** HIV, Men who have sex with men, Self-diagnosis

## Abstract

**Background:**

We assessed to what extent HIV self-testing would be incorporated by men who have sex with men (MSM) with previous testing history as their exclusive testing option and describe what actions they would take in the case of obtaining a reactive self-test.

**Methods:**

We conducted an online survey among Spanish resident MSM recruited mainly in gay dating apps and analyze 6171 ever tested individuals. We used Poisson regression to estimate factors associated with the incorporation of self-testing as the exclusive testing option. Among those who would incorporate self-testing as their exclusive option, we described actions taken if obtaining a reactive self-test by number of tests in the past.

**Results:**

Nearly half of the participants (48.3%) were > =35 years old, 84.6% were born in Spain, 57.9% had attained a university degree, 55.1% lived in a municipality of ≤500.000 and 86.4% self-identified as homosexual. For 37.2%, self-testing would become their exclusive testing option. The incorporation of self-testing as the exclusive option increased with age 25–34 (PR:1.1, 95%CI:1.0–1.3), 35–44 (PR:1.3, 95%CI:1.2–1.5), 45–49 (PR:1.5, 95%CI:1.3–1.7) and > 50 (PR:1.5, 95%CI:1.3–1.8) and in those who reported unprotected anal intercourse (PR:1.1, 95%CI:1.0–1.2) or having paid for sex (PR:1.2, 95%CI:1.0–1.3) in the last 12 months. It was also associated with having had < 10 HIV test in the past (2–9 tests (PR:1.3, 95%CI:1.1–1.4); 1 test (PR:1.5, 95%CI:1.3–1.7)), and having been tested ≥2 years (PR:1.4, 95%CI:1.3–1.5) or between 1 and 2 years ago (PR:1.1, 95%CI:1.0–1.2). Of participants who would use self-testing exclusively 76.6% would confirm their result in case of obtaining a reactive self-test and only 6.1% wouldn’t know how to react. Only one individual expressed that he would do nothing at all.

**Conclusion:**

HIV self-testing could become the exclusive testing option for more than a third of our participants. It was chosen as the exclusive option especially by older, at risk and under-tested MSM. Self-testing strategies need to especially consider the linkage to care process. In this sense, only a small fraction would not know how to react and virtually nobody reported taking no action if obtaining a reactive result.

**Supplementary Information:**

The online version contains supplementary material available at 10.1186/s12889-020-09976-9.

## Background

HIV remains an important public health problem in Western European countries such as Spain. Men who have sex with men (MSM) are the most affected group and comprised 56.4% of the total number of new HIV diagnoses reported in Spain during 2018 [1]. Promoting HIV testing and prompt linkage to care allows early treatment initiation and is a key element in the fight against the HIV epidemic. It not only reduces morbidity and mortality for those diagnosed with HIV [2, 3] but also helps to control the epidemic by reducing infectiousness from one person to another [4, 5].

Current recommendations are that MSM should test at least once every 12 months and even more frequent if having unprotected sex with occasional partners [6]. However, delayed diagnosis (CD4 count <350mm^3^) among MSM is still frequent (40.3%) [1] and estimates suggest that 18.8% of the MSM living with HIV in Spain are unaware of their infection [7]. In Spain, testing is offered free of charge in all levels of the national health system and it can also be found anonymously in a network of Sexual Health Clinics. Thanks to technological advances in HIV diagnosis, during the last decade a number of initiatives have taken testing outside of clinical settings. Thus, rapid testing can now be found in a number of community based organizations across Spain [8] and, in some regions, it is also offered in community pharmacies [9, 10]. The last testing strategy that has been incorporated into the array of already existing options is HIV self-testing (HIV-ST) which is legally available in the country since 2018 [11].

Self-testing is a convenient way of checking ones’ serostatus as it requires no appointments, no waits, and no pre and post-test discussion all of which have been described as barriers to testing [12]. HIV-ST could also alleviate barriers related to confidentiality [12]. On the other hand, its limitations are especially related to those obtaining a reactive result. If reactive, a self-test needs to be confirmed and can increase the risk of suboptimal linkage to care. Additionally, no face-to-face counselling is available for persons in the need of coping with a reactive self-test [13–16].

Previous studies conducted in Spain and other European countries have described that MSM have a modest knowledge of the existence of HIV-ST [17, 18] but tend to be supportive of it [19, 20] and report a high willingness to use it [18–20]. Nevertheless, a recent large European online study, [21] suggests that self-testing is still a residual option in Spain and in the rest of the European continent. Given its recent introduction, this could change in the coming years and HIV-ST is expected to gain popularity [22]. Until now, no one has studied the way in which self-testing will be incorporated by MSM into their current testing habits and we do not know if HIV-ST will completely replace on-site testing and become their exclusive testing option. The alteration of testing habits needs to be taken into account when introducing this new testing option. In this sense, one of the main worries surrounding HIV-ST is suboptimal linkage to care (timely confirmation testing and Anti-retroviral Therapy initiation) but very few studies have studied it [23] and actions taken by individuals who obtain a reactive self-test need to be carefully considered.

The aim of the present paper is to analyze self-testing’s potential of being incorporated as the exclusive testing option among MSM with previous testing experience and describe what would be the actions taken by those facing a reactive result. It is hypothesized that a substantial proportion of MSM will report that they would incorporate HIV-ST as their exclusive testing option and that most would seek confirmatory testing.

## Methods

### Study procedures

For the present study, we used data collected in a Spanish cross-sectional online survey that recruited MSM between September 2012 and April 2013. Potential respondents were invited to participate through banner advertisements located mainly in gay dating websites. Banners were showed to potential participants accessing from Spain. Those who clicked on it, were directed to the online questionnaire. Before beginning the survey, they had to give their acceptance in the consent form page where participants were informed about the purpose of the study, the time needed to complete the questionnaire and about the complete anonymity and confidentiality of the data collected. In this sense, no Internet Protocols or cookies were collected and to further secure the protection of respondent confidentiality no location information or date of birth were collected. Instead, participants were asked to report the approximate number of inhabitants of the area they lived in and their age in years. The study was approved by the Carlos III health institute ethical committee (CEI PI 70_2015).

### Study participants

Given that the size of our population (MSM who used the recruitment websites) was unknown, we used the most recent estimation of the number of MSM living in Spain (*N* = 386.800) [24] for sample size calculation. Since no previous studies were available to offer an approximated prevalence of our main outcome variable, we assumed a response distribution of 50%. Considering a margin of error of +/− 2% as acceptable, the minimum recommended size of our sample should be 2387.

The inclusion criteria for the present study were having being designated male at birth, being ≥18 years, having had sex with another man at least once and self-reporting HIV negative serostatus. Of the 9349 MSM who met the inclusion criteria we additionally excluded 2872 who had never been tested for HIV, 9 with unknown data of testing history and 387 that did not answer the main outcome question. Thus, the final sample was comprised of 6171 individuals.

### Data collection instrument

The self-administered questionnaire was designed in Spanish (see additional file 1). Before dissemination, a draft version was shared with several experts on the field who critically reviewed its content. The suggested changes were discussed and incorporated onto the questionnaire by the research team. Afterwards, the questionnaire was piloted in a reduced sample of gay men. They received a link to the questionnaire and were asked to fill it in and send back their feed-back. Based on their comments, modifications were introduced were needed. The responses given by participants in the pilot stage were not included in the analysis. During the initial days of recruitment, responses were analyzed to double check that no programming mistakes had been done.

The questionnaire was comprised of six domains that assessed the following areas:

- Sociodemography: Among other variables, we assessed age, country of birth, level of education and approximate number of inhabitants in municipality of residence during the past twelve months.
- Sexual orientation/outness: We assessed participant’s sexual orientation, level of involvement with the gay scene and disclosure of their sex life with other men.
- Previous HIV testing history: Participants were asked about the number of tests received in the past, time since last test and setting of last testing episode.
- STI history: Participants were asked if they had been diagnosed with an STI in the past and when did the last diagnosis occur.
- Risk Behaviors: We assessed lifetime sex with men and women, number of unprotected anal intercourses (UAI) with other men and transactional sex (paid or sold sex) during the last 12 months.
- HIV ST: This domain included questions that assessed the type of use that participants would give to HIV ST if approved and reactions following a hypothetical reactive result. At the of beginning this domain, a standardized definition of HIV ST was presented to all participants to ensure informed response. Type of use given to HIV ST was assessed with a close ended question with four answer options; (1) “I would never use it and I would always seek for testing performed by a professional”, (2) “I would only use it occasionally but most of the times I would seek for testing performed by a professional”, (3) “Self-testing would be my most frequent testing option although I would occasionally also seek for testing performed by a professional”, (4) “I would use self-testing exclusively; I do not think I would seek for testing anywhere else”. For those reporting that self-testing would become their exclusive testing option, we assessed reactions following a hypothetical reactive test.

### Statistical analysis

We performed a descriptive analysis of the main demographic and behavioral characteristics of all participants. We grouped quantitative variables to summarize their frequencies and relative frequencies.

The type of use that participants would give to HIV-self-testing kits was recoded into two categories to create our main outcome variable (exclusive use of HIV-ST /non-exclusive use of HIV-ST). We considered that self-testing would become the exclusive testing option for those choosing option 4: “I would use self-testing exclusively; I do not think I would seek for testing anywhere else”. To examine factors associated with reporting that self-testing would become their exclusive testing option, we calculated crude and adjusted prevalence ratios (PRs) and their corresponding 95% confidence intervals (CIs). We performed univariable and multivariable analysis using Poisson regression with robust variance since it is a better option in cross-sectional studies with frequent outcomes [25, 26]. Statistically significant factors at *p* < 0.1 in the univariable analysis were introduced in the multivariable regression model. To achieve model comparisons and select the optimal model we used the minimum Akaike information criteria (AIC) values. We conducted the analysis using STATA 11 software (StataCorp, Texas, United States, United States).

To summarize reactions following a hypothetical reactive HIV-ST, we stratified results by the number of lifetime HIV testing (1/> 1), and calculated the chi-square statistic.

## Results

The demographic and risk behavior characteristics along with the HIV testing practices of the 6171 participants included in the analysis are summarized in Table 1. The majority of them (68.5%) were 25–44 years old, 84.6% were born in Spain and 57.9% had completed university studies. One-third (33.7%) of the sample reported no involvement with the gay scene, 86.4% self-identified as homosexual, only 6.5% had not disclosed their sexual orientation to someone else and 58.8% only had had sex with men. During the previous twelve months, 33.3% of the participants reported > = 2 UAI, 47.3% had been diagnosed with an STI, 6.9% had paid and 4.7% had been paid for sex. Overall, 78.9% had received > 1 test and 44.7% reported having been tested in the preceding 12 months. Regarding the place where the last HIV test took place General Practitioner (39.3%) was the most frequently reported setting, followed by Sexual Health Clinics (21.2%).

**Table 1:** Main characteristics and HIV testing practices of 6171^a^ ever tested men who have sex with men

	N	%
**Age group**		
< 25	818	13.3
25–34	2367	38.4
35–44	1861	30.1
45–49	610	9.9
≥ 50	515	8.3
**Place of birth**		
Spain	5219	84.6
Latin-America	555	9.0
Other regions	397	6.4
**Education level**		
Primary/Secondary	771	12.5
Higher Secondary	1806	29.3
University	3576	57.9
**Municipality (number of inhabitants)**^**b**^		
≤ 500.000	3266	55.1
> 500.000	2656	44.9
**Relationship with gay culture**		
Member of a gay CBO^c^	387	6.6
Frequents gay scene but is not member of a gay CBO	2766	47.1
Only frequents gay scene to hook up with other men	736	12.5
Not related to gay scene in any way	1980	33.7
**Sexual orientation**		
Homosexual	5189	86.4
Hetero-Bisexual	814	13.6
**Has disclosed his sexual orientation to no one**	389	6.5
**Has had sex only with men (ever)**	3631	58.8
**Received an STI diagnosis**^**b**^	2814	47.3
**No. of unprotected anal intercourse**^**b**^		
0	2279	38.2
1	1704	28.5
≥ 2	1988	33.3
**Has paid for sex**^**b**^	416	6.9
**Has been paid for sex**^**b**^	284	4.7
**No. of previous HIV tests**		
≥ 10	814	13.2
2–9	4054	65.7
1	1303	21.1
**Time since last HIV test (years)**		
< 1	2758	44.7
1–2	1358	22.0
≥ 2	2055	33.3
**Setting of last HIV test**		
General practitioner	2423	39.3
Sexual health clinics	1308	21.2
Hospital or clinic during an admission	875	14.2
Private laboratory	646	10.5
HIV-AIDS related CBO	482	7.8
Employer or insurance company clinic	187	3.0
Others	247	3.9
**Way of using of self-testing If approved**		
Would never use self-testing	1608	26.1
Would use in combination with other testing options	2268	36.8
Would be my exclusive testing option	2295	37.2

^a^Due to missing data the numbers might not add up to totals

^b^In the last 12 months

^c^Community-Based Organization

Some 26.1% reported that they would never use self-testing if made available, 36.8% that they would use it in combination with other testing options and 37.2% that it would be their exclusive testing option. (Table 1).

In the univariable analysis, the demographic and lifestyle characteristics associated with using self-testing as the exclusive testing option are presented in Table 2. In the multivariable analysis (Table 2), the variables that remained significantly associated with the main outcome were age groups 25–34 (PR:1.1, 95%CI:1.0–1.3), 35–44 (PR:1.3, 95%CI:1.2–1.5), 45–49 (PR:1.5, 95%CI:1.3–1.7) and over 50 (PR:1.5, 95%CI:1.3–1.8), having had > = 1 UAI in the last 12 months (PR:1.1, 95%CI:1.1–1.2) and having paid for sex during the same time period (PR:1.2, 95%CI:1.1–1.3). It was also independently associated with reporting < 10 tests in the past (2–9 tests (PR: 1.3, 95%CI: 1.1–1.4); one test (PR: 1.5, 95%CI: 1.3–1.7)) and having been tested > = 1 year ago (≥2 years ago (PR: 1.4, 95%CI: 1.3–1.5); 1–2 years ago (PR: 1.1, 95%CI: 1.0–1.2)).

**Table 2:** Prevalence and associated factors to using HIV self-testing as the exclusive testing option if made available among ever tested MSM (*N* = 6171)

	Would exclusively use self-testing to test for HIV (***N*** = 2295)						
	n	% (row)	cPR	95% CIs	p	aPR	95% CIs
**Age**							
< 25 years old	250	30.6	1.0			1.0	
25–34	818	34.6	1.1	1.0–1.3	0.040	1.1	1.0–1.3
35–44	719	38.6	1.1	1.1–1.4	< 0.001	1.3	1.2–1.5
45–49	266	43.6	1.2	1.2–1.6	< 0.001	1.5	1.3–1.7
≥ 50 years old	242	47.0	1.2	1.3–1.8	< 0.001	1.5	1.3–1.8
**Education level**							
University	1305	36.5	1.0				
Primary/Secondary	309	40.1	1.0	1.0–1.2	0.057		
Higher Secondary	677	37.5	1.0	1.0–1.1	0.475		
**Sexual orientation**							
Homosexual	1903	36.7	1.0				
Hetero-Bisexual	327	40.2	1.1	1.0–1.2	0.050		
**Relationship with gay culture**							
Member of a gay CBO^b^	133	34.4	1.0				
Frequents gay scene but is not member of a gay CBO	985	35.6	1.0	0.9–1.1	0.634		
Only frequents gay scene to hook-up with other men	301	40.9	1.2	1.0–1.4	0.036		
Not related to gay scene in any way	757	38.2	1.1	0.9–1.3	0.160		
**Disclosure of sexual orientation**							
Has disclosed his sexual orientation to no one	166	42.7	1.0				
Has disclosed his sexual orientation to someone	2063	36.8	0.9	0.8–0.9	0.016		
**No. of unprotected anal intercourse**^a^							
0	806	35.4	1.0			1.0	
≥ 1	1405	38.1	1.1	1.0–1.2	0.038	1.1	1.1–1.2
**Paid for sex**^a^							
No	2049	36.5	1.0			1.0	
Yes	191	45.9	1.3	1.1–1.4	< 0.001	1.2	1.1–1.3
**No. of previous HIV tests**							
≥ 10	232	28.5	1.0			1.0	
2–9	1483	36.6	1.3	1.1–1.4	< 0.001	1.3	1.1–1.4
1	580	44.5	1.6	1.4–1.8	< 0.001	1.5	1.3–1.7
**Time since last HIV test (years)**							
< 1	847	30.7	1.0			1.0	
1–2	474	34.9	1.1	1.1–1.3	0.006	1.1	1.0–1.2
≥ 2	974	47.4	1.5	1.4–1.7	< 0.001	1.4	1.3–1.5

*cPR* crude prevalence ratio; *aPR* adjusted prevalence ratio; *CI* confidence interval

^a^In the last 12 months

^b^Community-Based Organization

Regarding the reactions following a reactive self-test reported by those who would use self-testing as their exclusive testing option, seeking a confirmatory test was the most frequently reported behavior: 33.2% would try to confirm the result in a health center and 30.6% would try to confirm it in an HIV/AIDS clinic and testing site (25.4% among those reporting one previous test and 32.3% in those with more than one test). Taking another self-test before making any decision and “not knowing how I would react in this situation”, was more frequently reported by one-time testers (19.4% vs. 13.9 and 8.8% vs. 5.2% respectively). Only one participant reported that he would do nothing at all in this situation. (Table 3).

**Table 3:** Most likely reaction following a reactive self-test result among those who reported that self-testing would be their exclusive testing option by number of lifetime HIV tests (*N* = 2295)

	Number of lifetime HIV testing						***P***-value
	1 (***N*** = 535)		> 1 (***N*** = 1652)		Total (***N*** = 2187)		
	n	%	n	%	n	%	0.001
I would try to confirm the result in a health center	175	32.7	550	33.3	725	33.2	
I would try to confirm the result in an HIV/AIDS clinic and testing site	136	25.4	533	32.3	669	30.6	
I would take another self-test before making any decision	104	19.4	229	13.9	333	15.2	
I don’t know how I would react in this situation	47	8.8	86	5.2	133	6.1	
I would try to confirm the result in a private laboratory	47	8.8	161	9.7	208	9.5	
I would try to confirm the result in an AIDS/Drug NGO counselling and testing site	16	3.0	56	3.4	72	3.3	
I would try to forget it and do nothing at all	0	0.0	1	0.1	1	0.0	
Other	10	1.9	36	2.2	46	2.1	

## Discussion

Our results suggest that HIV-ST could become the exclusive way of testing for more than a third of an online sample of MSM living in Spain. It would be used exclusively especially by older individuals, with UAI, who paid for sex and among those with less testing experience and longer time since last test. The majority, would seek for confirmatory testing in a variety of settings in the case of obtaining a reactive result.

In Europe, recent studies have shown that the potential of HIV-ST of becoming a valuable alternative to promote HIV diagnosis among untested MSM is high [19, 27, 28]. However, to our knowledge, this is the first time someone has investigated how HIV-ST would be incorporated to the already existing landscape of testing options.

The results of the multivariable analysis suggest that HIV-ST would be adopted as the exclusive testing option, especially by older individuals, those with higher number of UAI, higher rates of past STI and those not meeting current testing recommendations. Late diagnosis is more frequent among older individuals [1] suggesting the existence of barriers to timely diagnosis and linkage to care that could be reduced with the introduction of HIV-ST. Likewise, the fact that those not meeting current testing recommendations (last test > 12 months ago) and those with lower number of previous tests also presented higher probabilities of reporting that HIV-ST would become their exclusive testing option, could suggest that these individuals are not totally comfortable with existing testing options and could switch to remote testing options. The increased probabilities of reporting that HIV-ST would become their exclusive option in higher risk participants, is in line with a UK based study that concluded that the existence of remote testing could increase testing frequency among a small group of especially at risk MSM who favored characteristics of remote testing better than those from healthcare settings [29].

Regarding the effect of the adoption of HIV-ST as an exclusive testing option, two scenarios are possible: if by using HIV-ST as the only option, time between tests is reduced then it could have the potential to reduce time from diagnosis to infection increasing all the associated benefits from both the individual and public health perspective. In this sense, it is promising that HIV-ST has proven its efficacy in increasing testing frequency in randomized control trials [30, 31] but its effectiveness in a real-life scenario still needs to be assessed.

The opposite could also happen leading to the second scenario: the exclusive use of HIV-ST could result in greater times between tests. Until now, HIV-ST is not distributed free of cost. Price has been pointed out as one of the main barriers [29] to access HIV-ST and in Spain is between 25 and 30 euros. Whether if this price could lead to longer times between tests for those only using this testing option, remains unknown and should be taken into account.

Something that also needs to be addressed is the fact that HIV-ST could lead to missed opportunities to diagnose other sexually transmitted infections (STI) if they replace testing in sexual health services and other clinical settings. In fact, a Randomized Controlled Trial by Katz et al. found a lower frequency of STI testing among participants in the HIV-ST arm [30]. STIs such as Gonorrhea, syphilis or Lymphogranuloma Venereum have been on the rise among MSM in Spain [32] and in the rest of western Europe [33–35]. In our sample, this is especially relevant since MSM who reported UAI and/or having paid for sex presented increased probabilities of incorporating HIV-ST as the exclusive testing option. Thus, if HIV-ST is incorporated as the exclusive testing option there should be ways of ensuring that testing frequency for other STIs, is maintained.

The incorporation of HIV-ST as an exclusive testing option also poses other challenges. As a remote testing option, it could be affected by higher time to linkage to care. In this sense, our results are promising; most of our participants self-reported that they would seek confirmatory testing. However, a minority of participants reported not knowing how to react in the case of receiving a positive result. Investigating the characteristics of this small group of individuals and analyzing their reasons for not seeking immediate confirmatory testing is necessary to design strategies to ensure they are optimally linked to care.

Results are not without limitations. In the present study, we are working on a hypothetical situation rather than observed behavior. There is, however, some evidence that suggests that answers based on hypothetical situations are able to predict actual behavior [36–38]. Participants included in the study were recruited mainly through gay dating websites. Although the use of these online platforms to socialize and meet sexual partners is common among MSM [39, 40], precautions should be made when generalizing our results to other MSM. In this sense, MSM reporting more sexual risk behaviors could be overrepresented in our sample as has been previously reported [41]. Because of the nature of the question that assessed our main outcome, MSM who reported no previous testing experience could not be included in the analysis but a study conducted in Spain showed that HIV ST could be the gateway to testing for a high percentage of never tested MSM [19].

Almost two years since its introduction, future studies should be conducted to assess if HIV ST has been adopted as an exclusive option by MSM. We need to determine the size and characteristics of this population and how the adoption of this testing option has affected their testing frequency for HIV but also for other STIs. We also need to conduct studies to ascertain how participants with a reactive result have been linked to care compared to other non-remote settings.

## Conclusion

Our data suggest that the introduction of HIV-ST could result in a change of current testing patterns especially among undertested, older and especially at risk MSM. The effect of its incorporation by these subgroups as their exclusive testing option should be monitored to assess its effect on testing frequency and other unwanted outcomes such as STI underdiagnosis and suboptimal HIV linkage to care.

## Supplementary Information

**Additional file 1.** Survey questions used for the study in English.

## Data Availability

The datasets used and/or analyzed during the current study are available from the corresponding author on reasonable request.

## Abbreviations

CIs: confidence intervals
HIV-ST: HIV self-testing
MSM: men who have sex with men
PRs: prevalence ratios
RCT: randomized controlled trial
STI: sexually transmitted infection
UAI: unprotected anal intercourses
