# Supplementary material for: The incorporation of HIV self-testing as an exclusive option among men who have sex with men in Spain: results of an online cross-sectional study
Source: BMC Public Health. 2020 Dec 4;20:1865. doi: 10.1186/s12889-020-09976-9 (PMC7718697; doi:10.1186/s12889-020-09976-9)
Supplement: Supplementary file 1 — Additional file 1. Survey questions used for the study in English. [file 12889_2020_9976_MOESM1_ESM.docx]

**S1 Appendix. Survey questions used in this study**

**Age**

**¿Cuántos años tienes?** ________

**¿How old are you?** ________

**Place of birth**

**¿Has nacido en España?**

Sí

No, en otro país 🡪 ¿Cual?

**Were you born in Spain?**

Yes

No, in a diffferent country 🡪 Which one?

**Educational level**

**¿Cuál es el máximo nivel de estudios que has completado?**

Ninguno o estudios primarios (hasta los 12 años)

Enseñanza secundaria de primera etapa o formación profesional de grado medio (el nivel que debe acabarse a los 16 años)

Enseñanza secundaria de segunda etapa o formación profesional de grado superior (el nivel que debe acabarse a los 18 años)

Estudios universitarios

Otros/no sé clasificarme: describe con detalle los estudios más altos que has acabado:

**Which is the highest educational level you have completed?**

None or primary education (education up to 12 years of age)

First level secondary or middle grade vocational training (the level that should be finished at 16 years of age)

Second level secondary or upper grade vocational training (the level that should be finished at 18 years of age)

University studies

Others: please specify

**Place of residence**

**¿En los últimos 12 meses, donde has video la mayor parte de este tiempo?**

En España 🡪 ¿En qué Comunidad Autónoma?

En otro país 🡪 ¿Cuál?

**Where have you lived during most of the last 12 months?**

In Spain🡪 In which autonomous region?

In another country🡪 Which?

**Settlement Size**

**¿Cuántos habitantes tiene la localidad en que has vivido en los últimos 12 meses**

**(aproximadamente)?**

Más de un millón

Entre 500 mil y un millón

Entre 100 mil y 500 mil

Entre 50 mil y 100 mil

Entre 10 mil y 50 mil

Menos de 10 mil

**How many inhabitants does the place you have lived most of the last 12 months have (approximately)?**

More tan 1.000.000

Between 500.000 and 1.000.000

Between 100.000 and 500.000

Between 50.000 and 100.000

Between 10.000 and 50.000

Under 10.000

**Sexual Orientation**

**¿Actualmente, con cuál de estas opciones te sientes más identificado?**

Heterosexual

Homosexual

Bisexual

otras especificar:

**Currently, which of the following options do you think better suits you?**

Heterosexual

Homosexual

Bisexual

Others (specify)

**Disclosure sexual orientation**

**¿A quiénes les has contado que has tenido relaciones sexuales con otros hombres?**

**Contesta “Sí” o “No” para cada una de las respuestas**

- Amigos/as Sí No

- Hermano/a Sí No

- Tu madre Sí No

- Tu padre Sí No

- Compañeros de trabajo Sí No

**With whom have you shared that you have had sex with other men?**

Answer "Yes" or "No" for each of the following options:

- Friends Yes No

- Siblings Yes No

- Your mother Yes No

- Your father Yes No

- Co-workers Yes No

**No. of previous HIV tests**

***Aparte de si has donado sangre, ¿cuántas veces TE HAN hecho la prueba del VIH?**

Mas de 20 veces

15 a 20 veces

10 a 15 veces

6 a 9 veces

3 a 5 veces

2 veces

Una vez.

Nunca.

**Besides blood donation, how many times have you been tested for HIV?**

More than 20 times

15 to 20 times

10 to 15 times

6 to 9 times

3 to 5 times

2 times

Once.

Never.

**Time since last HIV test (years)**

***¿Cuánto hace que TE HICIERON la prueba del VIH por última vez?**

Hace /__/___/ (selecciona la unidad de tiempo)

**When was the last time you were tested for HIV?**

/__/___/ (select time unit) ago

**Setting of last HIV test**

**¿Dónde te la realizaron?**

Un hospital o clínica, durante un ingreso

Un centro de planificación familiar

Consulta del médico especialista

Médico de cabecera/ centro de salud

Laboratorio privado

Una farmacia

Urgencias de un hospital

Centro de enfermedades de transmisión sexual o de diagnóstico del VIH/sida

Servicios médicos de empresa o centro de trabajo

ONGs de sida o drogas (en su sede o en unidad móvil)

Otro sitio. Especificar cuál........................................................

**Where were you tested?**

A hospital or clinic, during an admission

A family planning center

Consultation of a specialist doctor

General practitioner / primary care center

Private laboratory

A pharmacy

Hospital emergencies

Sexual health clinic

Company or workplace medical services

AIDS or drug NGOs (at their headquarters or in a mobile unit)

Other site. Specify which one

**Sexual partners (lifetime)**

**El término "relaciones sexuales" puede aplicarse a conceptos diversos. Aquí consideramos SÓLO aquellas en las que hay penetración vaginal, anal u oral.**

**Teniendo en cuenta la definición anterior a lo largo de tu vida ¿Con quién has tenido relaciones sexuales?**

Nunca he tenido relaciones sexuales, ni con hombres ni con mujeres

Sólo con mujeres

Más a menudo con mujeres, pero al menos una vez con un hombre

Igual con hombres que con mujeres

Más a menudo con hombres, pero al menos una vez con una mujer

Sólo con hombres

**The term “sexual relations” can apply to different concepts. Here, we consider ONLY those with vaginal, anal or oral penetration**

**According to this definition ¿With whom have you ever had sexual relations?**

I have never had sex neither with men or women

Only with women

More often with women but at least once with another man

Equally with men and women

More often with men but at least one time with a woman

Only with men

**Relation with gay culture**

**Dinos cuáles de las siguientes situaciones describen tu relación con la cultura y el ambiente gay.**

Señala TODAS las opciones que correspondan

Soy miembro o colaboro con una asociación gay

Suelo salir por locales de ambiente gay con amigos/as

Sólo acudo a locales de ambiente gay para ligar

Casi nunca he estado en locales de ambiente gay

**Which of the following situations best ddescribes your rleationship with the gay scene and culture.**

I am a member or collaborate with a gay association

I usually hang out in gay venues with my friends

I only go to gay venues to hook-up with someone

I’ve hardly been to gay venues.

**Unprotected sex**

**¿Y aproximadamente con cuántos tuviste relaciones ANALES SIN preservativo en este periodo? (en los últimos 12 meses)**

Con ninguno

Uno

Dos

3-4

5-9

10-19

20-50

Más de 50

**And with how many did you have unprotected anal intercourse? (in the last 12 months)**

None

One

Two

3-4

5-9

10-19

20-50

Over 50

**STI diagnosis**

**¿Alguna vez te han diagnosticado una enfermedad de transmisión sexual? Ejs: gonococia, sífilis, clamidia, herpes genital, tricomonas, etc.**

Sí

No

**¿Cuánto tiempo hace que tuviste esta última enfermedad?**

Menos de 12 meses

Hace más de un año pero menos de 5

Hace 5 años o más

**Have you ever been diagnosed with a sexually transmitted infection such as gonococci, syphilis, genital herpes, chlamydia, trichomonas etc.?**

Yes

No

**When was the last one diagnosed?**

Less than 12 months ago

Between 1 and 5 years ago

5 or more years ago

**Transactional sex**

**Durante los ÚLTIMOS 12 MESES, ¿has pagado para tener relaciones sexuales?**

Sí

No

**Y ¿te han pagado por tenerlas?**

Sí

No

**In the LAST 12 MONTHS, have you been paid in exchange of sex?**

Yes

No

**And, have you paid for sex?**

Yes

No

**Reaction following a positive self-test**

**Si te hicieras el AUTOTEST y el resultado fuera positivo, ¿cómo crees que actuarías después?**

Me haría otro autotest antes de tomar cualquier decisión

Iría a un centro para que me hicieran otra prueba distinta para confirmar ese resultado positivo

Trataría de olvidarme del tema y no hacer nada más

No sé lo que haría en una situación así. No sé cómo reaccionaría

**¿En qué lugar te harías esa otra prueba?**

En un centro de salud

En un centro de planificación familiar

Laboratorio privado

Una farmacia

Centro de enfermedades de transmisión sexual o de diagnóstico del VIH/sida

Servicios médicos de empresa o centro de trabajo

ONGs de sida o drogas (en su sede o en unidad móvil)

Otro sitio. Especificar cuál:

**If you perform a self-test and the result happened to be positive ¿How do you think you would act?**

I would perform an additional self-test before making any decision

I would go to a center to take a confirmation test

I would try to forget about it

I am not sure what would I do.

**Where would you take the confirmation test?**

A primary care center

A family planning service

A private laboratory

A pharmacy

An anonymous medical center for the diagnosis of HIV and other STIs

A medical service at work

An NGO (at their office or in a mobile unit)

Others: please specify

**Frequency of use if self-test kits were already available**

**Imagina que el auto test empezara ya a venderse en las farmacias. ¿Cuál de las siguientes frases resume mejor la forma en que lo emplearías?**

Yo no lo utilizaría nunca, iría siempre a que me la hicieran los profesionales

Sólo lo utilizaría alguna que otra vez, la mayoría de las veces iría a que me hicieran la prueba

Sería la forma habitual de realizármela, aunque alguna vez iría a que me la hicieran

Sería la forma habitual de realizármela y no creo que fuera a que me la hicieran, salvo que el autotest me diera positivo

**Imagine that self-testing kits began to be marketed in pharmacies right now, Which of the following statements would represent the use you would make of them?**

I would never use it, I would always leave testing in the hands of a professional

I would only use it occasionally, I would normally go somewhere to get tested

Self-testing would be my most frequent testing option although I would occasionally also seek for testing performed by a professional

I would use self-testing exclusively; I do not think I would seek for testing anywhere else
